# Lipid Metabolism in Bovine Oocytes and Early Embryos under In Vivo, In Vitro, and Stress Conditions

**DOI:** 10.3390/ijms22073421

**Published:** 2021-03-26

**Authors:** Fabiana de Andrade Melo-Sterza, Ralf Poehland

**Affiliations:** 1 Laboratory of Reproduction Biotechnologies, Animal Science, State University of Mato Grosso do Sul, 79200-000 Aquidauana, Brazil; 2 Leibniz Institute for Farm Animal Biology, Institute of Reproduction Biology, 18196 Dummerstorf, Germany

**Keywords:** fatty acids, follicular fluid, complex cumulus–oocyte, mitochondria, heat stress, metabolism, oocyte, blastocyst

## Abstract

Lipids are a potential reservoir of energy for initial embryonic development before activation of the embryonic genome and are involved in plasma membrane biosynthesis. Excessive lipid droplet formation is detrimental to cryotolerance and is related to alterations in mitochondrial function, which likely affects lipid metabolism. Increased lipid accumulation in in vitro produced embryos is a consequence of the stress during in vitro embryonic development process. There are several open questions concerning embryo lipid metabolism and developmental potential. Oocyte maturation and embryo development in vivo and in vitro may vary if the donors are subjected to any type of stress before follicle puncture because crucial changes in oocyte/embryonic metabolism occur in response to stress. However, little is known about lipid metabolism under additional stress (such as heat stress). Therefore, in this review, we aimed to update the information regarding the energy metabolism of oocytes and early bovine embryos exhibiting developmental competence, focusing on lipid metabolic pathways observed under in vivo, in vitro, and stress conditions.

## 1. Introduction

The maturation and differentiation processes in oocytes and embryos are accompanied by biosynthesis as well as extensive reorganization of cellular compartmentalization processes that demand high energy consumption. Due to the presence of a relatively extensive apparatus with energy reserves in the form of lipid droplets and a multitude of mitochondria, the oocytes are well prepared to handle the high energy demand for maturation. However, the mechanisms that regulate metabolism take place in a very complex manner, linking important reactions that occur in follicular fluid, cumulus cells, and oocytes. Along with the regulation of oocyte energy reserves, the regulation of the energy supply is critical. Regulation of the activity and possibly the redistribution of mitochondria is important for maintaining the spatial and functional reorganization of lipid droplets as an energy source. However, the cellular biological processes are dependent on a multitude of cellular biochemical and molecular biological mechanisms, each with different energy requirements. The different processes must be precisely regulated in terms of the timing of the potential developmental capacity of the embryo. Different stresses, such as heat, influence the developmental capacity of oocytes and embryos in vivo [1].

Despite all the improvements in in vitro biotechniques in recent years, we should assume that the in vitro conditions of maturation, fertilization, and embryo cultivation alone are already serious stresses for oocytes and embryos. Adaptations to artificial culture conditions, in particular the culture media, already impose requirements that do not occur in such a way in vivo [2]. This is even more true for techniques such as intracytoplasmic sperm injection (ICSI), somatic nuclear transfer (SNT), or blastomere sampling, which require additional micromanipulation, bypass biological processes, or impede embryonic development. Ultimately, IVM bypasses the biological process of selecting ovulating follicles. Available biotechniques are used to varying extents in different species on a target-specific basis. While ICSI is a widely used technique in the human field, normal IVP, including cryopreservation, clearly dominates in the bovine field. Mice have been used in research for all these techniques. Completely different aspects and, above all, greater problems arise in the use of such techniques in wild and zoo animals, especially in endangered species [3,4]. In this review, we focused on bovine species.

Three aspects of energy or lipid metabolism are of particular interest. First, the oxygen tension in the in vitro culture is relevant; serum addition or serum replacement (chemically defined media) is important as a substrate source as well as potential regulatory substances, and there are some media additions in this context that are intended to influence the lipid balance. For decades, the optimization of in vitro embryo production has been studied with the aim of achieving the highest possible rate of developmentally competent embryos, leading to a high rate of healthy offspring. One of the most remarkable achievements in the IVP trajectory was the reduction in the incidence of large calf syndrome (LOS), which was probably caused by the reduction or exclusion of serum from the culture medium and by performing in vitro culture at low oxygen tension [5]. The first studies reporting LOS demonstrated the altered energy metabolism of embryos produced in vitro, drawing attention to the higher lipid content of these embryos [6].

The higher lipid content in the in vitro-produced embryos has been related to its low cryotolerance [7]; however, the use of delipidant agents during in vitro culture does not necessarily result in increased cryotolerance [8]. In addition to the stress of in vitro production, it is relevant to mention that the cumulus–oocyte complexes (COC) have their own history when they arrive in the laboratory, which influences subsequent in vitro development.

Heat stress, for example, is a very common type of stress that affects oocyte and embryo development. However, little is known about how lipid metabolism influences the embryonic development of oocytes under heat stress. However, it may be a good example to elucidate the importance of lipid reserves in oocytes and embryos under stress.

There are several unaddressed questions regarding embryo metabolism and developmental potential. Therefore, in this review, we aimed to update the knowledge on the energy metabolism in oocytes and early bovine embryos in terms of developmental competence, with a focus on the lipid metabolic pathways under in vivo, in vitro, and stress conditions.

## 2. Cumulus–Oocyte-Complex Lipid Metabolism

COCs contain two different cell types with distinct metabolic profiles and requirements. Oocytes mainly undergo oxidative phosphorylation, and cumulus cells (CCs) exhibit a high rate of glycolytic activity [9].

Metabolic crosstalk between oocytes and CC occurs through the bidirectional exchange of small molecules, including adenosine triphosphate (ATP), ions, and oxidative substrates, such as pyruvate and free fatty acids (FFA) [10].

Since there is a high demand for proteins during development, these substrates are unlikely to be used for ATP production and thus the endogenous stores of glycogen and protein are insufficient to sustain the developmental process [11].

Lipids are an important source of energy and may serve as the main substrate in porcine and bovine oocytes [12]. Fatty acid oxidation can generate approximately 3.5 more ATP molecules than glucose; therefore, it is a very efficient source of energy [13].

Energy from FFAs is produced via mitochondrial fatty acid oxidation (FAO). Carnitine palmitoyltransferases (CPT1 and CPT2), and their cofactor (carnitine) are critical for FFA transport into the mitochondria to undergo FAO, and thereafter, to produce ATP [14].

During oocyte maturation, among other metabolic pathways, active lipid synthesis, transport, storage, and degradation occur in the oocyte and surrounding CCs [10]. After maturation, a decrease in the activity of lipase (lipolysis) is observed in CCs, but no difference is observed in the activities of phosphofructokinase (glycolysis), and G6PDH (pentose phosphate pathway). During the growth stage and at the start of bovine oocyte maturation, CCs supply fatty acids to the oocyte, resulting from lipase activity [9]. Therefore, lipid metabolism in CCs affects oocyte survival and maturation success [10,15].

Inhibition of FAO promotes the death of CCs and prevents oocyte meiotic progression [10]. It has been suggested that triacylglycerol (TAG) species synthesized from FFA are supplied by CCs to the oocyte in the intact COC during maturation, as these lipids are not detected in the maturation medium, fetal bovine serum, immature CCs, and oocytes. Sanchez-Lazo et al. [10] also showed that the lipid profile of CCs varied based on the source of lipids used in the IVM medium. These observations indicate a role for CCs in regulating the synthesis and/or consumption of TAG by the oocyte to provide the energy required for the maturation process [15]. 

Homologous gap junctions between granulosa cells (GCs) and heterologous gap junctions between GCs and oocytes mediate their metabolism. Small molecules (<1 kDa), such as ATP, glucose, alanine, glycine, lysine, histidine, cysteine, and glutathion, might be directly transferred from GC/CC through gap junctions to the oocyte. Heterologous gap junctions, such as connexins 37 and 43, are essential for normal oocyte growth and maturation [16].

The transport of fatty acids from CCs to the oocytes might occur via transzonal projections (TZPs) and be mediated by fatty acid-binding protein 3 (FABP3). In vitro-matured COCs exhibit higher transcript and protein levels of FABP3 in CCs than in immature CCs obtained from in vivo matured COCs. FABP3 is localized within TZPs in immature and 9 h-matured COCs. However, after the first polar body extrusion, when TZPs are disconnected from the ooplasm, FABP3 molecules accumulate at the terminals of these projections. Moreover, they show simultaneous accumulation of FABP3 and lipids in the oocytes during the first 9 h of IVM and a reduction in lipid accumulation when TZPs were disrupted by cytochalasin B [17].

## 3. Oocytes

It is known that the oocytes of different mammalian species have different lipid contents, both in absolute terms (mouse, 4 ng; pig, 156 ng), and in relation to the ooplasm (mouse 2.5 × 10^−5^ ng/µm^3^; pig 25.9 × 10^−5^ ng/µm^3^) [18]. In addition, there are differences in the required maturation time of oocytes in vitro (murine 14 h, porcine 40–44 h, bovine 20–24 h), or in the developmental time after fertilization to the maternal-embryonic transition or blastocyst stage in these species [19]. A correlation between these two phenomena seems conceivable, as does the hypothesis that differences in energy metabolism between species are associated with them. Thus, the conclusions made here for cattle cannot be applied one-to-one to the other species, and vice versa. Oocytes derived from cows contain ~5.69 × 10^−5^ ng/µm^3^ fatty acids [11], and almost 50% (~2.3 × 10^−5^ ng/µm^3^) are stored in the form of lipid droplets (LD) [18]. Considering the high lipid content, the oocytes derived from cows, pigs, and sheep are visually darker than those derived from mice or humans [18]. 

It is widely accepted that oocytes with brown and homogeneous ooplasm surrounded by compact multi-layered cumulus stock are suitable for IVM. However, different patterns of ooplasma color have been observed, and can be used as a marker of superior embryo development. For example, oocytes with a homogenously distributed brown color with a black periphery and brown/black granulate ooplasma are considered to be developmentally fit. Three patterns of brown ooplasma exhibited high accumulation of lipids and good developmental potential, along with a high density of organelles, high polar body extrusion rate, and intermediate levels of ATP before IVM culture at the germinal vesicle (GV) stage. After IVM, the ATP levels of mature oocytes were higher than those of GV oocytes, indicating the intensity of metabolism during maturation [20].

Mitochondria are essential for supporting early embryonic development because glycolysis is limited during oocyte maturation and early embryonic development. Mitochondrial replication begins in primordial germ cells and continues during early oogenesis. During the later stages of folliculogenesis, the number of mitochondria increases significantly [21], from less than 10 in pre-migratory germ cells to 200 in oogonium, 6000 in primary oocytes, and more than 100,000 during cytoplasmic maturation [22,23]. However, there is no de novo synthesis of mitochondria that occurs between metaphase II and the blastocyst stage [21].

It has been demonstrated that immature bovine oocytes exhibit low mitochondrial activity [24], probably because mitochondria are immature at this point in time [25], thereby confirming that energy must be provided to the oocytes by the GCs at this developmental stage [26]. However, after maturation, oocytes exhibit a large percentage of mitochondria with high activity [24]. Additionally, mitochondrial activity is especially high in oocytes with better morphological quality and competence, wherein changes in the distribution pattern (in particular, the aggregation pattern of mitochondria in the cytoplasm), and high ATP production are observed [27,28,29]. The association between increased mitochondrial activity and changes in their distribution can be described not only in cattle but also in other species (e.g., pig: [30]; horse: [31]).

However, there is a limitation to increasing mitochondrial activity, as extremely high levels of ATP in matured oocytes may indicate impaired developmental competence and disruption of the regulation of mitochondrial functions in oocytes [12], suggesting that the storage of ATP at suitable levels in matured oocytes is a key factor in determining subsequent embryonic development and the quality of the resulting blastocysts. 

LD are an important substrate for energy storage and are also involved in the maintenance of membranes [18]. LD appear in the oocyte (80 µm) in early tertiary follicles of approximately 1 mm, and the number of LD increases gradually from the tertiary to the late tertiary follicle stage when the oocytes reach a size of >110 µm [32]. LD are composed of a neutral TAG core and a single phospholipid layer, often associated with various protein inclusions [18], such as the perilipin adipophilin tail-interacting protein family. PLIN 2 and PLIN 3 belong to the PAT family. PLIN2 prevents lipid degradation, thereby promoting lipid accumulation [33], and PLIN3 is associated with nascent LD when cells are challenged with an environment of high lipid levels [34]. Therefore, considerable variations in LD areas can be observed during maturation [35]. Approximately 15 h after attaining a luteinizing hormone (LH) peak, the number and size of LD increases and the mitochondria assemble around the droplets of the MI oocyte, which are then evenly distributed throughout the ooplasm. Approximately 24 h after attaining an LH peak, the MII oocyte generated from an ovulatory follicle, the LD, and the mitochondria reach a more central location [32]. A close spatial distribution of the endoplasmic reticulum (ER), mitochondria, and LD suggests a functional relationship between them in cattle oocytes [36]. LD may exhibit a dynamic distribution and relation to other organelles in the cell, which might change in accordance with the amount of FA they are submitted to [37]. 

**Figure 1:**
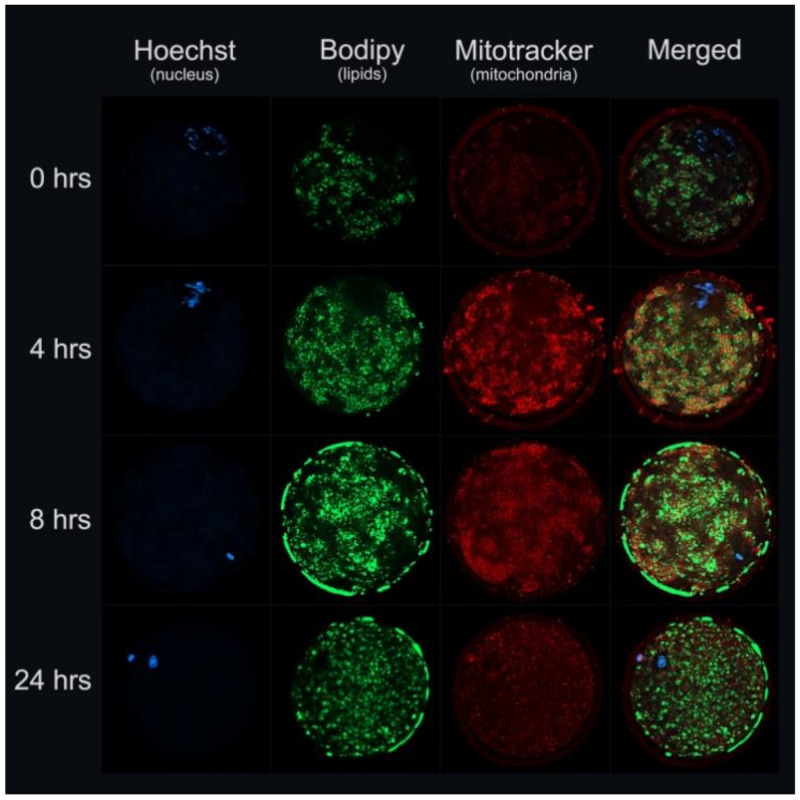
Illustrative images of bovine oocytes during in vitro maturation (0, 4, 8, and 24 h of maturation) demonstrating meiotic maturation (nucleus configuration, Hoechst 33,342-blue; Sigma-Aldrich, Merck KgaA, Darmstadt, Germany), lipid content (Bodipy 493/503-green, Molecular Probes, Eugene, OR, EUA), and mitochondrial activity (MitoTracker^®^ Orange-red, Molecular Probes, Eugene, OR, EUA) (vital staining, confocal laser scanning microscopy, Cardoso/Pöhland/Melo-Sterza, FBN-Lab).

Recently, we showed that the lipid content of *Bos taurus* oocytes increased in the first 4 h and remained similar until 24 h during IVM [38]. In Figure 1, we illustrate bovine oocytes during in vitro maturation (0, 4, 8, and 24 h of maturation) demonstrating meiotic maturation, lipid content, and mitochondrial activity. 

The oocyte has a mechanism to prevent the depletion of lipid stocks due to the elevated energy requirements during early cleavage, which is probably mediated by PLIN2, as its expression is upregulated before and after the maturation stage in bovine oocytes [39]. As mentioned earlier, the storage of fatty acids in LD protects the oocytes from lipotoxicity, and if the mechanisms involved in LD formation/maintenance are impaired, oocyte maturation will also be impaired. For example, the loss of FABP3 and FABP7 (both of which are involved in FA uptake), or PLIN2 leads to an increase in the NADP/NADPH ratio, and reactive oxygen species (ROS) levels during hypoxia, which leads to cell death [40]. Although FFA β-oxidation is essential for promoting adequate oocyte development and the benefits of lipids and LD for oocyte maturation are well known, a remarkably high amount of LD can induce damage, resulting in low-quality embryos. Lipid accumulation is more evident in in vitro than in vivo maturated oocytes, where the lipid content is similar to that in immature oocytes [17]. Presumably, lipid accumulation in IVM oocytes is a consequence of the stress conditions in which COCs are subjected in vitro. *Bos taurus* oocytes showed a higher number of LD than *Bos indicus* oocytes, as did oocytes matured in medium supplemented with fetal bovine serum. It has been demonstrated that embryos from oocytes with a higher lipid accumulation also have a higher number of LD [41]. Due to the relationship between lipid content and cryotolerance, different strategies have been studied, such as the reduction and/or replacement of fetal bovine serum in the culture media [41] and supplementation with delipidating agents [42], among others. Although these strategies often result in reduced lipid content, improvements in cryotolerance are not always achieved. Thus, it is still unclear what the right amount of lipid is that is compatible with the best quality embryo and with the highest cryotolerance. 

The lipid profile of the LD is also very important. It has been demonstrated that high-quality oocytes exhibit high levels of oleic acid, whereas in low-quality oocytes, there is a high level of stearic acid [36]. Therefore, it is necessary to improve the quality of oocytes and embryos by altering their lipid profiles by manipulating the diet or the composition of the maturation/cultivation media needs to be considered.

A well-established consequence of IVM is the failure of synchronization between nuclear and cytoplasmic maturation. Therefore, some research groups are working on developing pre-maturation systems for increasing the time for adequate cytoplasmic maturation. For example, the addition of 3-isobutyl-1-methylxanthine (IBMX) and forskolin (FSK) to maintain high levels of intracellular cAMP can prevent meiotic resumption, increase the competence of oocytes, and enrich the blastocyst rate [43,44]. High oxygen consumption, mitochondrial activity, and ATP levels as well as increased expression levels of genes involved in mitochondrial functions relative to oxidative phosphorylation were observed in oocytes after treatment with FSK and IBMX [44]. The lipid content of oocytes in the pre-IVM group was higher than that in the control. In addition, modifications in the gene expression of GCs after pre-maturation are associated with an increase in the developmental competence of bovine oocytes [43]. Conversely, the lipid content of blastocysts originating from the pre-IVM group was lower than that from the control (without pre-maturation). Additionally, an increase in the abundance of 10 unsaturated membrane lipids was observed in these blastocysts, which could be related to their improved quality [43]. 

## 4. Follicular Fluid

Follicular fluid (FF) composition may contain biomarkers for oocyte competence and embryo implantation potential [45]. The lipids in FF were suggested to be predictive of genetic merit for fertility in Holstein cows [46].

Previously, it was shown that human oocytes failed to cleave when FF exhibited decreased levels of lactate and choline/phosphocholine, increased levels of glucose and high-density lipoprotein (HDL), as well as high concentrations of total saturated fatty acids and low concentrations of total polyunsaturated fatty acids [47]. 

The lipid composition of blood serum and FF is positively correlated [48]. FFA within the blood serum enters the follicular fluid, which surrounds the developing oocyte and CCs, and is then taken up by the oocyte [18]. This probably occurs via membrane transporters, such as CD36 and SLC27A [10]. 

FF contains both HDL and FFA. HDL is the predominant source of cholesterol for granulosa and theca cell steroidogenesis and ovarian angiogenesis [49,50]. The most prevalent FFAs found in the serum and FF of high-yielding dairy cows are oleic, palmitic, and stearic acids [48]. However, it is important to emphasize that elevated concentrations of saturated fatty acids in the plasma and FF can impair cell survival by inducing the apoptotic signaling cascade [51], a phenomenon characterized by lipotoxicity. 

Lipotoxicity is commonly observed in high-producing cows during the negative energy balance and in obese women, who consequently show high concentrations of FFA in FF. This phenomenon can be mimicked by the addition of saturated fatty acids such as palmitic, steric, and/or oleic acid to the in vitro maturation medium [52,53,54], resulting in oxidative stress, ER stress, and mitochondrial stress, with consequent stimulation of unfolded protein response (UPRer and UPRmt). The UPRs stimulate the additional production of chaperones to combat stress; however, under severe stress, the UPR induces programmed cell death. The addition of unsaturated fatty acids, such as α-linolenic acid [53] or mitochondria-targeted antioxidants, such as Mitoquinone [54], can minimize the effects of lipotoxicity and improve in vitro embryo production rates.

A primary defense system of the body against lipotoxicity is the storage of FFA in LD; therefore, it is possible to observe higher numbers of LD in oocytes from follicular fluid rich in fatty acids [55]. Likewise, not only the concentration but also the lipid profile of FF plays an important role in oocyte competence. For example, competent bovine oocytes exhibit significantly lower levels of palmitic acid (C16:0) and total FFA, and significantly higher levels of linolenic acid (C18:3n3) compared to FF from the oocytes that fail to form blastocysts [56] (Table 1). No difference was observed in the concentration of estrogen, progesterone, and testosterone in the bovine FF of yielding oocytes that failed to develop or that reached the blastocyst stage in vitro [56]. Moreover, human FF derived from follicles aspirated for IVP, from which the oocyte resulted in blastocysts and pregnancy after embryo transfer, have been shown to exhibit high levels of phosphatidic acid, triacylglycerol, and phosphatidylglycerol [45] (Table 1).

Recent studies have shown that bovine follicles larger than 8 mm have a greater ability to develop expanded blastocysts at D7 and their FF, and the embryos produced after IVF, have a higher lipid content. Higher levels of glucose, ROS, glutathione, and superoxide dismutase activity, and lower levels of triglycerides were also identified in FF from larger follicles. These results suggest intense lipid metabolism, since there is mobilization of triglycerides and high availability of glucose (a precursor of acetyl-CoA, which in turn is a precursor of triglycerides) to replenish the mobilized lipids. Lipid metabolism in FF seems to provide a favorable oxidant–antioxidant profile [59]. Short exposure to elevated levels of FFA in FF during final maturation in response to cow fasting provided a negative energy balance (NEB) similar to that observed post-partum. NEB results in massive lipid accumulation in cumulus cells, but lipid accumulation is not observed in oocytes because of the protection of CC. The lower lipid accumulation observed in oocytes could be attributed to the high levels of mobilized oleic acid (C18:1) observed in FF, which in combination with the induced lipid storage in CCs, may protect the oocyte against lipotoxicity [51].

## 5. Embryos

All mammalian species seem to use pyruvate, fatty acids, and amino acids as sources of energy for embryo development [18]. As in oocytes, mitochondrial activity is downregulated during early embryonic development; however, it is sufficient to meet the energy requirements with minimal production of ROS. This is in accordance with the “Goldilocks principle” [60], which suggests that the best embryos are the most energy-efficient in response to an optimal range of metabolic activity, corresponding to neither very low nor very high activity. In bovine embryos, to meet the energy requirement of the first cleavage, ATP is produced mainly through the uptake of pyruvate and lactate through the TCA cycle [61]. Oxygen consumption and ATP requirement increase as the embryo approaches the blastocyst stage [62], which is mainly due to the requirement for energy to form the blastocoel and to support protein synthesis necessary for embryo mass development [60]. Along with pyruvate, glucose consumption also increases significantly, although only a small amount is oxidized through the TCA cycle for ATP production [61]. The embryonic capacity for glycogen synthesis as an energy source for development is established from the 16-cell stage embryo, when an increase in the phosphorylation of glycogenic GSK-3a/b is observed, which reaches the highest level at the blastocyst stage [63]. Parallel to the increased glucose consumption in the metabolism of a blastocyst, increased production of lactate is also observed. Considering that embryonic development occurs under oxygenation, both in vivo (about 5% O_2_) and in vitro (5 or 20% O_2_), this phenomenon can be characterized as an “aerobic glycolysis” which defines the Warburg Effect [64]. Aerobic glycolysis is a less efficient mechanism for ATP production, but its importance will be discussed further. Additionally, it is important to highlight that this effect is more evident in embryos produced in vitro than in embryos produced in vivo or by superovulation in mice [64]. 

The lipid profiles of embryos during the first cleavage stage exhibit several similarities. However, at the 8- to 16-cell stage, when the bovine embryonic genome is activated, the lipid profile of the embryo is highly changed, which can be correlated to the activation of mechanisms that regulate lipid metabolism to prepare for its transition to the morula and blastocyst stages. Corroborating this information, it has been shown that a significant increase in LD occurs in the morula stage followed by a decrease in the blastocyst stage, a stage in which the demand for lipids is high [65]. 

The lipid content of IVP embryos is higher than that of their in vivo derived (IVD) counterparts. Abe et al. [66] showed that serum-containing medium, in contrast to serum-free medium, leads to abnormal accumulation of LD in early embryos and to problems after cryopreservation. The authors showed that after performing IVC in the presence of serum, embryos are produced with higher amounts of giant LD (>6 µm) and high levels of immature mitochondria [66]. In addition, Del Collado et al. [67] demonstrated that fetal calf serum in maturation medium leads to an up to 18-fold higher lipid concentration in oocytes compared to serum albumin, but in contrast to Abe et al. [66], both del Collado et al. and Choi et al. [67,68] found no changes in blastocyst or pregnancy rates after embryo transfer [68]. Furthermore, del Collado et al. [67] assumed in the same paper an influence on mitochondrial activity in an in vitro system. This is supported by other studies [69], which describe a correlation between higher mitochondrial activity in a subpopulation of matured oocytes with higher lipid content.

To avoid the use of fetal bovine serum (FBS) supplementation in IVC medium, several alternatives have been tested [65,70,71]. For example, the replacement of FBS with different concentrations of bovine serum albumin (BSA) results in better cryosurvival and differential relative abundance of developmentally important gene transcripts compared to embryos cultured in serum-containing media [70]. In another example, FBS was replaced with 0.05% sericin as a protein supplement during IVC, which reduced lipid content, but did not increase the re-expansion and hatching rates after vitrification/warming; however, the total cell number of blastocysts was highly similar to that of IVD embryos [71]. Until now, the composition of IVC medium, especially in terms of the protein source, has not been standardized among laboratories, which makes it difficult to compare different studies and results at the commercial level.

The comparison between IVP and IVD embryos is the best model for studying embryo quality. The lipid content of *Bos taurus* IVP and IVD embryos was higher than that of *Bos indicus* IVP and IVD embryos [58]. These differences between sub-species and embryo origin are supported by the differences observed in the lipid profile of the membrane and intracytoplasmic LD, gene expression, and cryotolerance among them [58,65]. Surprisingly, despite exhibiting high lipid content, *Bos taurus* embryos are more cryotolerant. In terms of the lipid profile, phosphatidylcholines (PC), especially PC (32:0), were less abundant and PC (34:2) was more abundant in IVD embryos of *Bos taurus*, thereby acting as negative and positive biomarkers for cryopreservation, respectively [58]. 

Different IVC supplementation strategies are being used to reduce the lipid content in bovine embryos, such as trans-10 cis-12 conjugated linoleic acid (CLA) and L-carnitine [8,42,72]. Although the lipid content in most cases was successfully reduced, it was not correlated with cryotolerance [8,72]. It is possible that the increase in lipid content is not causal for low cryotolerance but is a consequence of stress [72]. Conversely, [42] observed a better cryosurvival in embryos cultured in the presence of L-carnitine; however, no significant difference was observed in the pregnancy rates between treated and untreated embryos. 

In vivo embryonic development occurs under low oxygen tension, with a balance of ROS and antioxidant substance production. When embryos are produced in vitro, especially at high oxygen tension (20%), an accumulation of ROS occurs, characterizing oxidative stress. Oxidative stress can produce harmful effects on embryonic development, and among these effects, we highlight metabolic alterations, such as the depletion of ATP levels, changes in ion channels, deleterious effects on protein synthesis, lipid peroxidation, alterations in membrane permeability, and alterations in mitochondrial and ER functions [73,74,75,76]. Recently, it was confirmed that the embryos produced in vitro under low oxygen tension are of better quality, and it was possible to show that these embryos present a higher metabolic activity, based on the upregulation of genes linked to cellular metabolism, especially lipids, sugars, and proteins [77].

Controversial results of blastocyst rates after L-carnitine (a cofactor of β-oxidation with antioxidant activity) supplementation during IVC were observed with respect to O_2_ tension [42,72,78,79]. Our research group hypothesized that the addition of 3.03 mM L-carnitine for 24 h during IVC under high oxygen tension would produce embryos of similar quality compared to those produced without L-carnitine under low oxygen tension. We observed that L-carnitine supplementation for 24 h was not sufficient to reduce lipid content, but IVC under low oxygen tension without L-carnitine produced embryos of better quality, which can be related to the nitric oxide concentration observed on day 9 of IVC. We hypothesized that the biological activity of NO, induced by low O_2_ tension, protected the embryos against ROS toxicity and thus provided a better environment for embryonic development until hatching [80].

Recently, it was demonstrated that the Warburg Effect could explain the improved quality and cryotolerance of embryos produced in vitro under low oxygen tension [81]. The authors demonstrated that the proteome of embryos produced at low oxygen tension showed upregulation of key proteins involved in glycolysis, pyruvate metabolism, fatty acid degradation, and inositol phosphate metabolism. The protein pattern was confirmed by the upregulation of genes involved in glucose transport (*GLUT3*), glycolysis intermediates/regulators, such as phosphofructokinase-1 (*PFK1*) and glyceraldehyde phosphate dehydrogenase (*GAPDH*), and in the conversion of pyruvate to lactate (*LDHA*). In addition, the higher cryotolerance observed in embryos produced under low oxygen tension was related to the lower accumulation of LD, which could be explained by the elevated expression of proteins involved in cholesterol synthesis (HMGCS1 and HMGCR), and fatty acid degradation (ACAT2 and ACSL4). The altered lipid metabolism observed may be related to increased fluidity of the plasma membrane of the embryo and a reduction in the number of cytoplasmic LD [81]. Thus, the importance of the Warburg Effect has been shown to involve much more than ATP production, since the intermediates and regulators of glycolysis generated by this reaction play an essential role in embryonic development. 

Variations in the expression of genes involved in lipid metabolism in preimplantation bovine embryos, such as *ACSL3*, *ELOVL5,* and *ELOVL6* [65], have been demonstrated in embryos at the morula and blastocyst stages. ACS (acyl-CoA synthetase) is an enzyme that activates complex lipid biosynthesis and beta-oxidation by adding the CoA group to the reaction. ACS has several isoforms that have specific functions based on their intracellular localization. ACSL3 is related to the formation of LD in embryos. Similarly, elongation of very-long-chain fatty acids (*ELOVL*) is associated with the embryonic membrane phospholipid composition [65], and *ELOVL5* modulates intracytoplasmic LD [82]. 

To exemplify the importance of these genes in embryonic development, we can cite a study that added a positive modulator of ACSL3 (GW3965 hydrochloride) during IVC. The authors observed an increase in the lipid content of the expanded blastocysts, which led to an increase in cryosurvival, especially in low-quality embryos when subjected to vitrification [83]. Another study showed an increase in cytoplasmic lipid droplet deposition after transient gene expression knockdown of *ELOVL5*. Interestingly, no apparent deleterious effect on embryonic development and blastocyst cell number was observed, but a reduction in the expression of specific lipid genes was observed [82], demonstrating that further studies need to be conducted to clarify how altered lipid metabolism does not interfere with embryo quality.

An approach considered as a marker of embryo quality is the analysis of the kinetics of embryo development. It is well demonstrated that embryos that undergo early cleavage are more likely to develop into blastocysts [57,84], and this approach should help us to understand the correlation between embryo quality and lipid metabolism.

**Figure 2:**
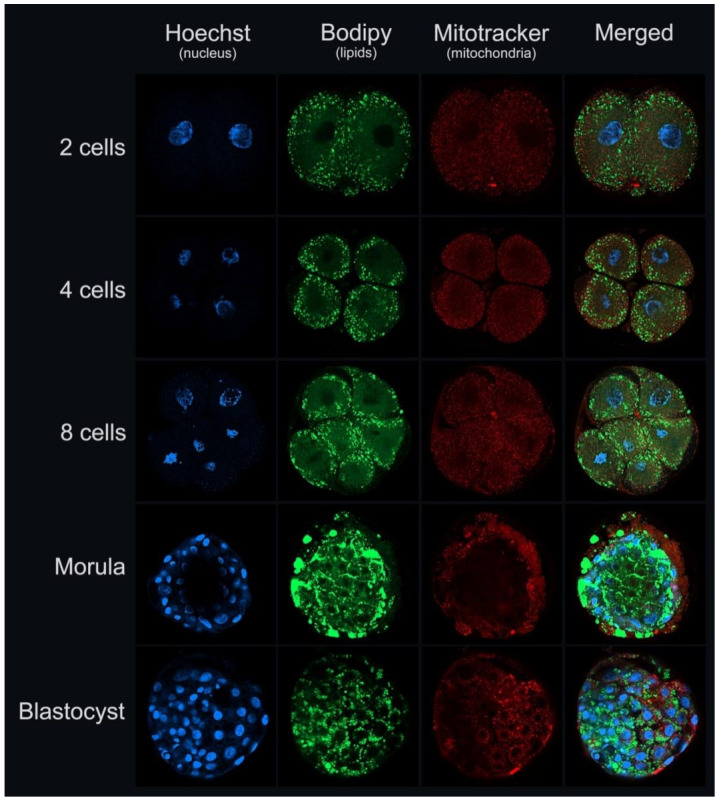
Illustrative images of bovine embryos during in vitro culture (2, 4, 8 cell stage, morula, and blastocyst), demonstrating cell number (nucleus staining, Hoechst 33,342–blue; Sigma-Aldrich, Merck KgaA, Darmstadt, Germany), lipid content (Bodipy 493/503–green; Molecular Probes, Eugene, OR, EUA), and mitochondrial activity (MitoTracker^®^ Orange–red; Molecular Probes, Eugene, OR, EUA) (vital staining, confocal laser scanning microscopy, Cardoso/Pöhland/Melo-Sterza, FBN-Lab).

Recently, we showed that blastocysts generated from earlier embryo cleavage exhibit higher lipid content than later embryo cleavage [85]. Additionally, another group showed that the lipid profile, especially of phosphatidylcholines, was different between fast- and slow-growing embryos at the 8–16 cell stage. Moreover, IVD blastocysts exhibit a different lipid profile to IVP blastocysts, but unlike the other group, no differences in the lipid profile of fast-and slow-growing IVP blastocysts were observed [57]. These findings indicate that the lipid content and profile of the embryo are correlated with its quality, but the underlying biological mechanisms are yet to be well understood. In Figure 2, we illustrate bovine embryos during in vitro cultivation (2, 4, and 8 cells, morula and blastocyst) demonstrating DNA, lipid content, and mitochondrial activity.

## 6. Tubal Fluids

The oviduct is the organ where fertilization and embryonic development occur until approximately the fourth day after fertilization in cattle. The composition of the oviduct fluid (OF) varies between estrous cycle phases and, if altered, may influence processes involving sperm capacitation, fertilization, and early embryonic development [86,87]. During the early luteal phase, OF is composed of cholesterol, triglycerides, non-esterified fatty acids (NEFA), proteins, glucose, and lactate. OF and plasma have similar concentrations of NEFA in healthy cows that probably alter the lipid composition of oviduct cells [88]. Alterations in the oviduct microenvironment can affect embryo development; thus, it is comprehensible that alterations in feed composition also affect pregnancy establishment. Considering the scope of this review, we will focus on the lipid composition of the OF. 

The oviduct contains a complex mixture of cholesterol, glycerophospholipids (phosphatidylinositol, PI; phosphatidylcholine, PC; phosphatidylethanolamine, PE; phosphatidylserine, PS), lysophospholipids (lysoPC, and lysoPE), sphingomyelins, and carnitines [87]. The origin and mechanisms involved in the accumulation of PL in the OF are not yet known, but they are believed to originate from cells of the oviduct epithelium and extracellular vesicles (EVs). The relationship between EVs and embryos could be verified after supplementation of the in vitro culture medium with EVs isolated from the oviduct of cows in the post-ovulatory period. It was demonstrated that embryos produced in the supplemented medium had a higher concentration of PC, SM, and PE of high molecular masses (PC 34:1, PC 36:4, and PC 36:3), than the medium not supplemented, similar to that of those found in the EVs [89]. 

The variation in the hormonal profile of females during the estrous cycle and its influence on animal behavior and physiology to establish and maintain pregnancy is well known. Recently, it was found that the lipid profile of the OF also varies throughout the estrous cycle and apparently occurs under endocrine regulation [87]. The participation of enzymes involved in the biosynthesis of phosphatidic acid (phospholipase c, diacylglycerol kinase, and phospholipase D2) in steroidogenesis reinforces the relationship between lipid metabolism and estradiol, a hormone present in high concentrations in the peri-ovulatory period [45]. It has been speculated that the lipid profile of the peri-ovulatory period is related to mechanisms that allow higher sperm fertility because spermatozoa are rich in long-chain fatty acids and are sensitive to changes in lipid profile in their environment [87]. Another example of the relationship between lipid profile and fertility and estradiol influence was demonstrated by Belaez et al. [57], who identified that cows with larger pre-ovulatory follicles and larger corpora lutea presented a higher pregnancy rate and a different uterine lipid profile than cows with lower fertility (Table 1).

## 7. Heat Stress

Exogenous stress, that is, stress acting on the animal system, usually has an indirect effect on oocytes and embryos, which is, therefore, more difficult to study. Extreme environmental conditions (i.e., heat stress), restrictive feeding, high physical strain, high milk production, and social stress are examples of exogenous stress that impact reproductive fitness. This phenomenon has been demonstrated in several studies [1,90]. However, the impact of these factors on the metabolism of oocytes, zygotes, and embryos is not yet understood. We assume that a large part of the different stress situations also primarily influences the energy metabolism of the animal. The mechanisms or the control points with which these imbalances are compensated or where adaptations occur could therefore be quite similar with regard to reproduction. 

Heat stress, for example, affects animals because the heat generated during metabolism in the body is not sufficiently released into the environment. The influence of heat stress is likely caused by the adaptation of the animal to this condition. This adaptation occurs to a large extent by attempting to minimize endogenous heat production, thereby reducing food consumption, milk production, and weight gain [91]. This is ultimately associated with a reduction in energy metabolism. These systemic effects can affect the regulation of energy metabolism in oocytes or embryos, but the underlying mechanism has not yet been elucidated. 

Our research group demonstrated some examples of how biological systems react to the adaptation process. For example, we showed that different tropical breeds [92] respond differently to the “stress” they are subjected to, especially under different housing conditions [93]. Additionally, the duration and intensity of in vitro thermal stress [94] exhibited an interesting pattern of oocyte and embryo development after maturation under cold and high temperatures. However, the biological processes involved are not yet completely understood, and we speculate that lipid metabolism can explain some of the open questions.

It is known that heat stress in the postpartum stage aggravates the NEB in high-yielding dairy cows. Despite the reduction in dominant follicle diameter, the biochemical concentrations of glucose, IGF1, urea, cholesterol, and NEFA are altered in the serum and FF of the dominant follicle. Specifically, total cholesterol was found to be lower, and NEFA levels were higher in cows under heat stress. All the alterations observed in FF after heat stress may result in inferior quality of oocytes and GCs [95].

Heat stress during the first 12 h of IVM hastened GVBD and GVBD is correlated with triglyceride and phospholipid content, independent of IVM temperature. In contrast, higher ATP levels were observed in heat-stressed oocytes after 24 h of in vitro maturation [96]. Under the experimental conditions of the aforementioned study, it was not possible to correlate the in vitro heat stress with lipolytic activity during oocyte maturation. However, the higher ATP production by oocytes matured under heat stress may be related to the increased beta-oxidation of fatty acids not detected in this study [96]. Additionally, bovine COCs subjected to heat stress during IVM exhibited transcriptional changes in oocytes that were related to changes in the electron transport chain and oxidative phosphorylation reactions. Moreover, mitochondrial disturbance and high ATP activity were observed in oocytes and early developing embryos, but not in blastocysts, probably because embryos with higher ATP content did not develop into blastocysts [96]. Therefore, there is a need for a better understanding of lipid metabolism under heat stress conditions.

## 8. Conclusions

Oocytes and cells in early-stage embryos rapidly differentiate. Therefore, several phenomena occur simultaneously, such as normal differentiation, which leads to the acquisition of fertilization competence, activation of the embryonic genome, differentiation into different cell lines, response to specific environmental conditions (stress and nutrition), and individual differences. The energy metabolism in oocytes and early embryos has a fundamental and significant impact on their developmental competence. Moreover, lipid metabolism, as the main source of energy and switch points to important regulatory pathways, is of central importance. Several factors (including in vitro vs. in vivo, exogenous stress, nutritional status) influence the quantity and composition of lipids. However, after many years of research, the mechanisms through which the lipid content and profile alter IVP embryo development are still not clear. It seems that the lipid profile is more likely to affect the embryo quality. This makes sense since certain lipids are also components of other pathways and a change in the lipid profile thus has an influence on developmentally relevant pathways in addition to the actual energy metabolism. Studies on junctions between energy metabolism and differentiation pathways (e.g., sirtuins) could provide new insights. Our knowledge, especially regarding the regulation and timing of changes in lipid metabolism, is limited. In in vivo conditions, the interaction between the oocytes with the intrafollicular milieu and the surrounding CCs appears to have a very significant impact on differentiation, timing, and synchronization. A simulation of these complex processes in vitro using currently available culture systems is not possible. In the field of basic research, three-dimensional culture models that have recently been developed and are ready for use, could help to recreate the in vivo conditions slightly better. Currently, however, we have to assume that the mechanisms underlying the regulation of lipid metabolism in vivo and in vitro may be distinct. Therefore, an efficient strategy to manipulate lipid metabolism has not been established to enhance the efficiency of IVP. Basic knowledge is still needed for further advances in the optimization of in vitro embryo production and related biotechniques in cattle and other species. Novel methodological approaches, especially the combination of dynamic-life cell studies using single-cell and downstream biochemical (e.g., lipid profiles) or molecular (e.g., RNA-seq) analyses might contribute to enhancing our knowledge in the near future.

## Figures and Tables

**Table 1:** Concentration of specific lipid subclasses in tubal fluids and embryos associated with higher fertility.

Lipids	Cell/Fluid	Association with Higher Fertility	Reference
Higher concentrations of the n-3 PUFA linolenic acid	Follicular Fluid	Oocyte competence—potential to develop to the blastocyst stage in vitro	[56] (bovine)
Higher concentrations of Phosphatidic acid (PA; 745.5563 m/z)	Follicular Fluid	Pregnancy probability	[45] (woman)
Higher concentrations of Triacylglycerol (TAG; 773.6153 m/z)	Follicular Fluid	Pregnancy probability	[45] (woman)
Higher concentrations of Phosphatidylglycerol (PG; 749.5693 m/z).	Follicular Fluid	Pregnancy probability	[45] (woman)
Lower concentrations of Glucosylceramide (GluCer) (796.6948 m/z)	Follicular Fluid	Pregnancy probability	[45] (woman)
Higher concentrations of Arachidonic acid (C20:4n6)	Follicular Fluid	Oocyte competence—potential of human oocyte to cleave.	[47] (woman)
Higher concentrations of Stearic acid (C18:0)	Follicular Fluid	Oocyte competence—potential of human oocyte to cleave.	[47] (woman)
Lower concentrations of palmitic acid (C 16:00)	Follicular Fluid	Oocyte competence—potential of bovine oocyte to develop to the blastocyst stage in vitro and of human oocyte to cleave.	[56] (bovine); [47] (woman)
Lower concentrations of total saturated fatty acids	Follicular Fluid	Oocyte competence—potential of bovine oocyte to develop to the blastocyst stage in vitro and of human oocyte to cleave.	[56] (bovine), [47] (woman)
Lower n-6:n-3 PUFA ratio	Follicular Fluid	Oocyte competence—potential of human oocyte to cleave.	[47] (woman)
Lower concentrations of Arachidic acid (C20:0)	Follicular Fluid	Oocyte competence—potential of human oocyte to cleave.	[47] (woman)
Lower concentration of Phosphatidylcholines (PC 36:4; 38:7; 38:5; 40:7; 40:6)	Uterus Fluid D4	Present in cows with bigger pre-ovulatory follicle and bigger corpus luteum	[57] (bovine)
Higher concentrations of Phosphatidylcholines (PC 32:0; 32:1; 34:4)	Uterus Fluid D4	Present in cows with bigger pre-ovulatory follicle and bigger corpus luteum	[57] (bovine)
Higher concentrations Ceramides (CER 42:1)	Uterus Fluid D4	Present in cows with bigger pre-ovulatory follicle and bigger corpus luteum	[57] (bovine)
Higher concentration of Phosphatidylcholines (PC 32:1; 35:2)	Uterus Fluid D7	Present in cows with bigger pre-ovulatory follicle and bigger corpus luteum	[57] (bovine)
Higher concentrations of Sphingomyelins (PC 34:2; 34:1)	Uterus Fluid D7	Present in cows with bigger pre-ovulatory follicle and bigger corpus luteum	[57] (bovine)
Higher concentration of Phosphatidylcholines—PC 34:2	Blastocyst	Potential for survival to cryopreservation	[58] (bovine)
Lower concentration of Phosphatidylcholines—PC 32:0	Blastocyst	Potential for survival to cryopreservation	[58] (bovine)

## Data Availability

Not applicable.

## Abbreviations

IVEP	in vitro embryo production
FF	follicular fluid
FFA	Free fatty acids
HDL	high-density lipoprotein
NEB	negative energy balance
COC	cumulus–oocyte complex
CC	cumulus cells
ATP	adenosine triphosphate
FAO	fatty acid oxidation
CPT	carnitine palmitoyltransferase
GC	granulosa cell
TZP	transzonal projections
FABP3	fatty acid-binding protein 3
LD	lipid droplet
TAG	triacylglycerol
FSK	forskolin
GV	germinal vesicle
PAT	Perilipin Adipophilin Tail-interacting
ER	endoplasmic reticulum
ROS	reactive oxygen species
FBS	fetal bovine serum
IVD	in vivo derived
IVP	in vitro-produced
BSA	bovine serum albumin
PC	phosphatidylcholines
CLA	conjugated linoleic acid
NEFA	non-esterified fatty acids
ICSI	intracytoplasmic sperm injection
SNT	somatic nuclear transfer

## References

[1] Hansen P.J. (2019). Reproductive physiology of the heat-stressed dairy cow: Implications for fertility and assisted reproduction. Anim. Reprod..

[2] Summers M.C., Biggers J.D. (2003). Chemically defined media and the culture of mammalian preimplantation embryos: Historical perspective and current issues. Hum. Reprod. Updat..

[3] Jewgenow K., Braun B.C., Dehnhard M., Zahmel J., Goeritz F. (2016). Research on reproduction is essential for captive breeding of endangered carnivore species. Reprod. Domest. Anim..

[4] Paris M.C.J., Mastromonaco G.F., Paris D.B.B.P., Krisher R.L. (2007). A perspective on the role of emerging technologies for the propagation of companion animals, non-domestic and endangered species. Reprod. Fertil. Dev..

[5] Ealy A.D., Wooldridge L.K., McCoski S.R. (2019). Board Invited Review: Post-transfer consequences of in vitro-produced embryos in cattle. J. Anim. Sci..

[6] Young L.E., Sinclair K.D., Wilmut I. (1998). Large offspring syndrome in cattle and sheep. Rev. Reprod..

[7] Sudano M.J., Paschoal D.M., Rascado T.D.S., Magalhães L.C.O., Crocomo L.F., De Lima-Neto J.F., Landim-Alvarenga F.D.C. (2011). Lipid content and apoptosis of in vitro-produced bovine embryos as determinants of susceptibility to vitrification. Theriogenology.

[8] Dias L.R.O., Leme L.O., Sprícigo J.F.W., Pivato I., Dode M.A.N. (2019). Effect of delipidant agents during in vitro culture on the development, lipid content, gene expression and cryotolerance of bovine embryos. Reprod. Domest. Anim..

[9] Cetica P., Pintos L., Dalvit G., Beconi M. (2002). Activity of key enzymes involved in glucose and triglyceride catabolism during bovine oocyte maturation in vitro. Reproduction.

[10] Sanchez-Lazo L., Brisard D., Elis S., Maillard V., Uzbekov R., Labas V., Desmarchais A., Papillier P., Monget P., Uzbekova S. (2014). Fatty Acid Synthesis and Oxidation in Cumulus Cells Support Oocyte Maturation in Bovine. Mol. Endocrinol..

[11] Ferguson E.M., Leese H.J. (2006). A potential role for triglyceride as an energy source during bovine oocyte maturation and early embryo development. Mol. Reprod. Dev..

[12] Sturmey R.G., Leese H.J. (2003). Energy metabolism in pig oocytes and early embryos. Reproduction.

[13] Dunning K.R., Cashman K., Russell D.L., Thompson J.G., Norman R.J., Robker R.L. (2010). Beta-Oxidation Is Essential for Mouse Oocyte Developmental Competence and Early Embryo Development1. Biol. Reprod..

[14] Bonnefont J.-P. (2004). Carnitine palmitoyltransferases 1 and 2: Biochemical, molecular and medical aspects. Mol. Asp. Med..

[15] Vireque A.A., Tata A., Belaz K.R.A., Grázia J.G.V., Santos F.N., Arnold D.R., Basso A.C., Eberlin M.N., Silva-De-Sá M.F., Ferriani R.A. (2017). MALDI mass spectrometry reveals that cumulus cells modulate the lipid profile ofin vitro-matured bovine oocytes. Syst. Biol. Reprod. Med..

[16] Collado-Fernandez E., Picton H.M., Dumollard R. (2012). Metabolism throughout follicle and oocyte development in mammals. Int. J. Dev. Biol..

[17] Del Collado M., Da Silveira J.C., Oliveira M.L.F., Alves B.M.S.M., Simas R.C., Godoy A.T., Coelho D.S.J., Marques A.B.M.D.S., Carriero M.M., Nogueira E.M. (2017). In vitro maturation impacts cumulus–oocyte complex metabolism and stress in cattle. Reproduction.

[18] Bradley J., Swann K. (2019). Mitochondria and lipid metabolism in mammalian oocytes and early embryos. Int. J. Dev. Biol..

[19] Santos R.R., Schoevers E.J., Roelen B.A.J. (2014). Usefulness of bovine and porcine IVM/IVF models for reproductive toxicology. Reprod. Biol. Endocrinol..

[20] Nagano M. (2019). Acquisition of developmental competence and in vitro growth culture of bovine oocytes. J. Reprod. Dev..

[21] Babayev E., Seli E. (2015). Oocyte mitochondrial function and reproduction. Curr. Opin. Obstet. Gynecol..

[22] Trimarchi J.R., Liu L., Porterfield D.M., Smith P.J., Keefe D.L. (2000). Oxidative Phosphorylation-Dependent and -Independent Oxygen Consumption by Individual Preimplantation Mouse Embryos. Biol. Reprod..

[23] Cummins J. (2004). The role of mitochondria in the establishment of oocyte functional competence. Eur. J. Obstet. Gynecol. Reprod. Biol..

[24] Tarazona A., Rodriguez J., Restrepo L., Olivera-Angel M. (2006). Mitochondrial Activity, Distribution and Segregation in Bovine Oocytes and in Embryos Produced in Vitro. Reprod. Domest. Anim..

[25] Tamassia M., Nuttinck F., May-Panloup P., Reynier P., Heyman Y., Charpigny G., Stojkovic M., Hiendleder S., Renard J.-P., Chastant-Maillard S. (2004). In Vitro Embryo Production Efficiency in Cattle and Its Association with Oocyte Adenosine Triphosphate Content, Quantity of Mitochondrial DNA, and Mitochondrial DNA Haplogroup. Biol. Reprod..

[26] Fair T. (2003). Follicular oocyte growth and acquisition of developmental competence. Anim. Reprod. Sci..

[27] Stojkovic M., Machado S.A., Stojkovic P., Zakhartchenko V., Hutzler P., Gonçalves P.B., Wolf E. (2001). Mitochondrial Distribution and Adenosine Triphosphate Content of Bovine Oocytes Before and After In Vitro Maturation: Correlation with Morphological Criteria and Developmental Capacity After In Vitro Fertilization and Culture1. Biol. Reprod..

[28] Jeseta M., Knitlova D.C., Hanzalova K., Hulinska P., Hanulakova S., Milakovic I., Nemcova L., Kanka J., Machatkova M. (2014). Mitochondrial Patterns in Bovine Oocytes with Different Meiotic Competence Related to Their in vitro Maturation. Reprod. Domest. Anim..

[29] Kątska-Książkiewicz L., Alm H., Torner H., Heleil B., Tuchscherer A., Ryńska B. (2011). Mitochondrial aggregation patterns and activity in in vitro cultured bovine oocytes recovered from early antral ovarian follicles. Theriogenology.

[30] Torner H., Brüssow K.-P., Alm H., Ratky J., Pöhland R., Tuchscherer A., Kanitz W. (2004). Mitochondrial aggregation patterns and activity in porcine oocytes and apoptosis in surrounding cumulus cells depends on the stage of pre-ovulatory maturation. Theriogenology.

[31] Torner H., Alm H., Kanitz W., Goellnitz K., Becker F., Poehland R., Bruessow K.-P., Tuchscherer A. (2007). Effect of Initial Cumulus Morphology on Meiotic Dynamic and Status of Mitochondria in Horse Oocytes during IVM. Reprod. Domest. Anim..

[32] Hyttel P., Fair T., Callesen H., Greve T. (1997). Oocyte growth, capacitation and final maturation in cattle. Theriogenology.

[33] Bickel P.E., Tansey J.T., Welte M.A. (2009). PAT proteins, an ancient family of lipid droplet proteins that regulate cellular lipid stores. Biochim. Biophys. Acta BBA Mol. Cell Biol. Lipids.

[34] Wolins N.E., Quaynor B.K., Skinner J.R., Schoenfish M.J., Tzekov A., Bickel P.E. (2005). S3-12, Adipophilin, and TIP47 Package Lipid in Adipocytes. J. Biol. Chem..

[35] Prates E.G., Nunes J.T., Pereira R.M. (2014). A Role of Lipid Metabolism during Cumulus-Oocyte Complex Maturation: Impact of Lipid Modulators to Improve Embryo Production. Mediat. Inflamm..

[36] Brusentsev E.Y., Mokrousova V.I., Igonina T.N., Rozhkova I.N., Amstislavsky S.Y. (2019). Role of Lipid Droplets in the Development of Oocytes and Preimplantation Embryos in Mammals. Russ. J. Dev. Biol..

[37] Valm A.M., Cohen S., Legant W.R., Melunis J., Hershberg J.M.U., Wait E., Cohen A.R., Davidson M.W., Betzig E., Lippincott-Schwartz W.R.L.E.B.J. (2017). Applying systems-level spectral imaging and analysis to reveal the organelle interactome. Nat. Cell Biol..

[38] Cardoso C.J.T., Melo-Sterza F.A., Drawert B., Poehland R. (2020). Lipid accumulation and mitochondrial activity during in vitro maturation of bovine oocytes. Reprod. Domest. Anim..

[39] Sastre D., Da Costa N.N., De Sá A.L.A., Conceição S.D.B., Chiaratti M.R., Adona P.R., Guemra S., Meirelles F.V., Santos S.D.S.D., Sena L. (2014). Expression of PLIN2 and PLIN3 during oocyte maturation and early embryo development in cattle. Theriogenology.

[40] Welte M.A., Gould A.P. (2017). Lipid droplet functions beyond energy storage. Biochim. Biophys. Acta Mol. Cell Biol. Lipids.

[41] Ordoñez-Leon E., Merchant H., Medrano A., Kjelland M., Romo S. (2014). Lipid Droplet Analysis Using In Vitro Bovine Oocytes and Embryos. Reprod. Domest. Anim..

[42] Zolini A.M., Carrascal-Triana E., De King A.R., Hansen P.J., Torres C.A.A., Block J. (2019). Effect of addition of l-carnitine to media for oocyte maturation and embryo culture on development and cryotolerance of bovine embryos produced in vitro. Theriogenology.

[43] Razza E.M., Sudano M.J., Fontes P.K., Franchi F.F., Belaz K.R.A., Santos P.H., Castilho A.C.S., Rocha D.F.O., Eberlin M.N., Machado M.F. (2018). Treatment with cyclic adenosine monophosphate modulators prior to in vitro maturation alters the lipid composition and transcript profile of bovine cumulus–oocyte complexes and blastocysts. Reprod. Fertil. Dev..

[44] Hashimoto S., Yamanaka M., Yamochi T., Iwata H., Kawahara-Miki R., Inoue M., Morimoto Y. (2019). Mitochondrial function in immature bovine oocytes is improved by an increase of cellular cyclic AMP. Sci. Rep..

[45] Montani D.A., Braga D.P.D.A.F., Borges E., Camargo M., Cordeiro F.B., Pilau E.J., Gozzo F.C., Fraietta R., Turco E.G.L. (2019). Understanding mechanisms of oocyte development by follicular fluid lipidomics. J. Assist. Reprod. Genet..

[46] Moore S.G., O’Gorman A., Brennan L., Fair T., Butler S.T. (2017). Follicular fluid and serum metabolites in Holstein cows are predictive of genetic merit for fertility. Reprod. Fertil. Dev..

[47] O’Gorman A., Wallace M., Cottell E., Gibney M.J., McAuliffe F.M., Wingfield M., Brennan L. (2013). Metabolic profiling of human follicular fluid identifies potential biomarkers of oocyte developmental competence. Reproduction.

[48] Leroy J.L.M.R., Vanholder T., Mateusen B., Christophe A., Opsomer G., De Kruif A., Genicot G., Van Soom A. (2005). Non-esterified fatty acids in follicular fluid of dairy cows and their effect on developmental capacity of bovine oocytes in vitro. Reproduction.

[49] Azhar S., Tsai L., Medicherla S., Chandrasekher Y., Giudice L., Reaven E. (1998). Human Granulosa Cells Use High Density Lipoprotein Cholesterol for Steroidogenesis. J. Clin. Endocrinol. Metab..

[50] Von Otte S., Paletta J.R.J., Becker S., König S., Fobker M., Greb R.R., Kiesel L., Assmann G., Diedrich K., Nofer J.-R. (2006). Follicular Fluid High Density Lipoprotein-associated Sphingosine 1-Phosphate Is a Novel Mediator of Ovarian Angiogenesis. J. Biol. Chem..

[51] Aardema H., Lolicato F., Van De Lest C.H., Brouwers J.F., Vaandrager A.B., Van Tol H.T., Roelen B.A., Vos P.L., Helms J.B., Gadella B.M. (2013). Bovine Cumulus Cells Protect Maturing Oocytes from Increased Fatty Acid Levels by Massive Intracellular Lipid Storage. Biol. Reprod..

[52] Lolicato F., Brouwers J.F., Van De Lest C.H., Wubbolts R., Aardema H., Priore P., Roelen B.A., Helms J.B., Gadella B.M. (2015). The Cumulus Cell Layer Protects the Bovine Maturing Oocyte Against Fatty Acid-Induced Lipotoxicity. Biol. Reprod..

[53] Marei W.F., De Bie J., Mohey-Elsaeed O., Wydooghe E., Bols P.E., Leroy J.L. (2017). Alpha-linolenic acid protects the developmental capacity of bovine cumulus–oocyte complexes matured under lipotoxic conditions in vitro. Biol. Reprod..

[54] Marei W.F.A., Bosch L.V.D., Pintelon I., Mohey-Elsaeed O., Bols P.E.J., Leroy J.L.M.R. (2019). Mitochondria-targeted therapy rescues development and quality of embryos derived from oocytes matured under oxidative stress conditions: A bovine in vitro model. Hum. Reprod..

[55] Warzych E., Pawlak P., Pszczola M., Cieslak A., Madeja Z.E., Lechniak D. (2017). Interactions of bovine oocytes with follicular elements with respect to lipid metabolism. Anim. Sci. J..

[56] Matoba S., Bender K., Fahey A.G., Mamo S., Brennan L., Lonergan P., Fair T. (2014). Predictive value of bovine follicular components as markers of oocyte developmental potential. Reprod. Fertil. Dev..

[57] Belaz K.R.A., Tata A., França M.R., Da Silva M.I.S., Vendramini P.H., Fernandes A.M.A., D’Alexandri F.L., Eberlin M.N., Binelli M. (2016). Phospholipid Profile and Distribution in the Receptive Oviduct and Uterus During Early Diestrus in Cattle. Biol. Reprod..

[58] Sudano M.J., Santos V.G., Tata A., Ferreira C.R., Paschoal D.M., Machado R., Buratini J., Eberlin M.N., Landim-Alvarenga F.D. (2012). Phosphatidylcholine and Sphingomyelin Profiles Vary in *Bos taurus* indicus and *Bos taurus* taurus In Vitro- and In Vivo-Produced Blastocysts. Biol. Reprod..

[59] Annes K., Sudano M.J., Belaz K.R.A., Tata A., Santos V.G., Junior A.M.D.F., Dos Santos É.C., Eberlin M.N., Milazzotto M.P. (2019). Lipid characterization of in vitro-produced bovine embryos with distinct kinetics of development. Zygote.

[60] Leese H.J., Guerif F., Allgar V., Brison D.R., Lundin K., Sturmey R.G. (2016). Biological optimization, the Goldilocks principle, and how much islagomin the preimplantation embryo. Mol. Reprod. Dev..

[61] Thompson J. (2000). In vitro culture and embryo metabolism of cattle and sheep embryos—A decade of achievement. Anim. Reprod. Sci..

[62] Leese H.J. (2012). Metabolism of the preimplantation embryo: 40 years on. Reproduction.

[63] García-Herreros M., Simintiras C.A., Lonergan P. (2018). Temporally differential protein expression of glycolytic and glycogenic enzymes during in vitro preimplantation bovine embryo development. Reprod. Fertil. Dev..

[64] Smith D.G., Sturmey R.G. (2013). Parallels between embryo and cancer cell metabolism. Biochem. Soc. Trans..

[65] Sudano M.J., Rascado T.D., Tata A., Belaz K.R., Santos V.G., Valente R.S., Mesquita F.S., Ferreira C.R., Araújo J.P., Eberlin M.N. (2016). Lipidome signatures in early bovine embryo development. Theriogenology.

[66] Abe H., Yamashita S., Satoh T., Hoshi H. (2001). Accumulation of cytoplasmic lipid droplets in bovine embryos and cryotolerance of embryos developed in different culture systems using serum-free or serum-containing media. Mol. Reprod. Dev..

[67] Del Collado M., Saraiva N.Z., Lopes F.L., Gaspar R.C., Padilha L.C., Costa R.R., Rossi G.F., Vantini R., Garcia J.M. (2016). Influence of bovine serum albumin and fetal bovine serum supplementation during in vitro maturation on lipid and mitochondrial behaviour in oocytes and lipid accumulation in bovine embryos. Reprod. Fertil. Dev..

[68] Choi B.-H., Park B.-Y., Kong R., Son M.-J., Park C.-S., Shin N.-H., Cheon H.-Y., Yang Y.-R., Lee J.-W., Jin J.-I. (2019). Effect of Serum and Serum Free Media on the Developmental Competence of OPU Derived Bovine IVP Embryo. J. Anim. Reprod. Biotechnol..

[69] Jeong W., Cho S., Lee H., Deb G., Lee Y., Kwon T., Kong I. (2009). Effect of cytoplasmic lipid content on in vitro developmental efficiency of bovine IVP embryos. Theriogenology.

[70] Rizos D., Gutiérrez-Adán A., Pérez-Garnelo S., De La Fuente J., Boland M., Lonergan P. (2003). Bovine embryo culture in the presence or absence of serum: Implications for blastocyst development, cryotolerance, and messenger RNA expression. Biol. Reprod..

[71] Hosoe M., Inaba Y., Hashiyada Y., Imai K., Kajitani K., Hasegawa Y., Irie M., Teramoto H., Takahashi T., Niimura S. (2016). Effect of supplemented sericin on the development, cell number, cryosurvival and number of lipid droplets in cultured bovine embryos. Anim. Sci. J..

[72] Held-Hoelker E., Klein S., Rings F., Salilew-Wondim D., Saeed-Zidane M., Neuhoff C., Tesfaye D., Schellander K., Hoelker M. (2017). Cryosurvival of in vitro produced bovine embryos supplemented with l -Carnitine and concurrent reduction of fatty acids. Theriogenology.

[73] Takahashi M., Keicho K., Takahashi H., Ogawa H., Schultz R.M., Okano A., Schulte R. (2000). Effect of oxidative stress on development and DNA damage in in-vitro cultured bovine embryos by comet assay. Theriogenology.

[74] Guerin P., El Mouatassim S., Ménézo Y. (2001). Oxidative stress and protection against reactive oxygen species in the pre-implantation embryo and its surroundings. Hum. Reprod. Updat..

[75] Cagnone G.L., Sirard M.-A. (2013). Transcriptomic signature to oxidative stress exposure at the time of embryonic genome activation in bovine blastocysts. Mol. Reprod. Dev..

[76] Yoon S.-B., Choi S.-A., Sim B.-W., Kim J.-S., Mun S.-E., Jeong P.-S., Yang H.-J., Lee Y., Park Y.-H., Song B.-S. (2014). Developmental Competence of Bovine Early Embryos Depends on the Coupled Response Between Oxidative and Endoplasmic Reticulum Stress. Biol. Reprod..

[77] Leite R.F., Annes K., Ispada J., De Lima C.B., Dos Santos É.C., Fontes P.K., Nogueira M.F.G., Milazzotto M.P. (2017). Oxidative Stress Alters the Profile of Transcription Factors Related to Early Development on In Vitro Produced Embryos. Oxidative Med. Cell. Longev..

[78] Takahashi T., Inaba Y., Somfai T., Kaneda M., Geshi M., Nagai T., Manabe N. (2013). Supplementation of culture medium with L-carnitine improves development and cryotolerance of bovine embryos produced in vitro. Reprod. Fertil. Dev..

[79] Ghanem N., Ha A.-N., Fakruzzaman M., Bang J.-I., Lee S.-C., Kong I.-K. (2014). Differential expression of selected candidate genes in bovine embryos produced in vitro and cultured with chemicals modulating lipid metabolism. Theriogenology.

[80] Pereira D.M., Cardoso C.J.T., Da Silva W.A.L., Souza-Cáceres M.B., Santos M., Pöhland R., Couto A.M., Moslaves I.S.B., Kadri M.C.T., Sterza F.D.A.M. (2020). Production of in vitro bovine embryos supplemented with l-carnitine in different oxygen tensions and the relation to nitric oxide. Zygote.

[81] Shahzad Q., Pu L., Wadood A.A., Waqas M., Xie L., Pareek C.S., Xu H., Liang X., Lu Y. (2020). Proteomics Analysis Reveals that Warburg Effect along with Modification in Lipid Metabolism Improves In Vitro Embryo Development under Low Oxygen. Int. J. Mol. Sci..

[82] Lanzarini F., Pereira F., Camargo J., Oliveira A., Belaz K., Melendez-Perez J., Eberlin M., Brum M., Mesquita F., Sudano M. (2021). ELOVL5 Participates in Embryonic Lipid Determination of Cellular Membranes and Cytoplasmic Droplets. Int. J. Mol. Sci..

[83] Valente R.S., De Almeida T.G., Alves M.F., De Camargo J., Basso A.C., Belaz K.R.A., Eberlin M.N., Landim-Alvarenga F.D.C., Fontes P.K., Nogueira M.F.G. (2019). Modulation of long-chain Acyl-CoA synthetase on the development, lipid deposit and cryosurvival of in vitro produced bovine embryos. PLoS ONE.

[84] Oliveira C.S., De Barros B.A.F., Monteiro C.A.S., Rosa P.M.S., Leal G.R., Serapião R.V., Camargo L.S.A. (2019). Individual assessment of bovine embryo development using a homemade chamber reveals kinetic patterns of success and failure to reach blastocyst stage. Syst. Biol. Reprod. Med..

[85] Cardoso C.J.T., Drawert B., Poehland R., Melo-Sterza F.A. (2020). Lipid content and mitochondrial activity of bovine embryos with different developmental kinetics. XXXIV Reunião Anual da Sociedade Brasileira de Tecnologia de Embriões, Animal Reproduction. Belo Horizonte: Brazilian College of Animal Reproduction. Anim. Reprod..

[86] Lamy J., Gatien J., Dubuisson F., Nadal-Desbarats L., Salvetti P., Mermillod P., Saint-Dizier M. (2018). Metabolomic profiling of bovine oviductal fluid across the oestrous cycle using proton nuclear magnetic resonance spectroscopy. Reprod. Fertil. Dev..

[87] Banliat C., Tomas D., Teixeira-Gomes A.-P., Uzbekova S., Guyonnet B., Labas V., Saint-Dizier M. (2019). Stage-dependent changes in oviductal phospholipid profiles throughout the estrous cycle in cattle. Theriogenology.

[88] Jordaens L., Van Hoeck V., De Bie J., Berth M., Marei W.F., Desmet K.L., Bols P.E., Leroy J.L. (2017). Non-esterified fatty acids in early luteal bovine oviduct fluid mirror plasma concentrations: An ex vivo approach. Reprod. Biol..

[89] Banliat C., Le Bourhis D., Bernardi O., Tomas D., Labas V., Salvetti P., Guyonnet B., Mermillod P., Saint-Dizier M. (2020). Oviduct Fluid Extracellular Vesicles Change the Phospholipid Composition of Bovine Embryos Developed In Vitro. Int. J. Mol. Sci..

[90] Nakamura K., Sheps S., Arck P.C. (2008). Stress and reproductive failure: Past notions, present insights and future directions. J. Assist. Reprod. Genet..

[91] Hansen P. (2004). Physiological and cellular adaptations of zebu cattle to thermal stress. Anim. Reprod. Sci..

[92] Souza-Cácares M., Fialho A., Silva W., Cardoso C., Pöhland R., Martins M., Melo-Sterza F. (2019). Oocyte quality and heat shock proteins in oocytes from bovine breeds adapted to the tropics under different conditions of environmental thermal stress. Theriogenology.

[93] Da Silva W.A.L., Poehland R., De Oliveira C.C., Ferreira M.G.C.R., De Almeida R.G., Cáceres M.B.S., Macedo G.G., Silva E.V.D.C.E., Alves F.V., Nogueira E. (2020). Shading effect on physiological parameters and in vitro embryo production of tropical adapted Nellore heifers in integrated crop-livestock-forest systems. Trop. Anim. Health Prod..

[94] Pöhland R., Souza-Cácares M.B., Datta T.K., Vanselow J., Martins M.I.M., Da Silva W.A.L., Cardoso C.J.T., Melo-Sterza F.D.A. (2020). Influence of long-term thermal stress on the in vitro maturation on embryo development and Heat Shock Protein abundance in zebu cattle. Anim. Reprod..

[95] Shehab-El-Deen M., Leroy J., Fadel M., Saleh S., Maes D., Van Soom A. (2010). Biochemical changes in the follicular fluid of the dominant follicle of high producing dairy cows exposed to heat stress early post-partum. Anim. Reprod. Sci..

[96] Hooper L.M., Payton R.R., Rispoli L.A., Saxton A.M., Edwards J.L. (2015). Impact of heat stress on germinal vesicle breakdown and lipolytic changes during in vitro maturation of bovine oocytes. J. Reprod. Dev..

